# Application of ILO ergonomic checkpoints for workplace health and safety assessment in post-conflict small and medium-sized enterprises in the Kurdistan Region of Iraq

**DOI:** 10.1038/s41598-026-41231-w

**Published:** 2026-03-11

**Authors:** Mohammed Qadir Ali, Omid Akbarzadeh, Rasoul Ahmadpour, Parisa Moshashaei, Parvin Sarbakhsh, Seyed Shamseddin Alizadeh

**Affiliations:** 1 College of Nursing, University of Raparin, Kurdistan Region, Rania, Sulaimani Government Iraq; 2 Department of Occupational Health and Safety, Faculty of Health, Tabriz University of Medical Sciences, Tabriz, Iran; 3 Health and Environment Research Centre, Tabriz University of Medical Sciences, Tabriz, Iran; 4 School of Built Environment and Design, Faculty of Art and Design, University of Canberra, Canberra, Australia; 5 Department of Epidemiology and Biostatistics, Tabriz University of Medical Sciences, Tabriz, Iran

**Keywords:** Workplace health and safety, Small and medium-sized enterprises, ILO ergonomic checkpoints, Post-conflict industrial settings, Occupational risk assessment, Participatory ergonomics, Occupational health, Public health

## Abstract

Workplace health and safety (WHS) in small and medium-sized enterprises (SMEs) is especially challenging in post-conflict regions with limited regulation. This study applies the ILO ergonomic checkpoints framework to assess WHS across five industrial sectors in the Kurdistan Region of Iraq. A cross-sectional study was performed among 70 SMEs, spanning food, metal, construction, furniture, and chemical industries. Ergonomic compliance data were collected using a structured observational checklist. Sectoral differences in safety performance were examined through ANOVA and ANCOVA, while correlation analysis explored the associations between WHS outcomes and organizational characteristics. Assumption checks confirmed statistical validity through Shapiro–Wilk tests, Q-Q plots, Levene’s test, and Variance Inflation Factors (VIF). The results demonstrated substantial disparities in ergonomic safety compliance across sectors, with food and construction enterprises exhibiting the lowest total safety scores. Among enterprise-level factors, managerial experience and workforce size were significantly associated with WHS performance, whereas average wage and company age showed weaker links. A conceptual predictive model is proposed to guide future research, positioning the total safety score as a function of key organizational variables. This study provides a validated ergonomics-based methodology and a conceptual model to guide WHS improvements in post-conflict SMEs. Findings support targeted interventions such as WHS training and participatory risk assessments. The model offers a scalable foundation for future predictive analytics and policy design in resource-limited settings.

## Introduction

### Background

Workplace health and safety (WHS) is increasingly recognized as a foundation of the Sustainable Development Goals, especially in developing and fragile economies^1^. In recent decades, WHS has emerged as a major global issue with various implications for public health^2^, economic development^3^, and basic human rights^4^. Low-income and developing countries undergoing industrialization have led to the expansion of informal sectors and small-scale enterprises, many of which operate in unsafe and unhealthy conditions in the absence of regulatory and government agencies^5^. WHS, which was previously an inter-organizational issue, is now recognized as a fundamental factor for sustainable development^6^ and decent work in developing and post-conflict regions^7^.

According to estimations by the International Labour Organization (ILO), more than 2.3 million workers lose their lives each year due to occupational accidents or work-related diseases, correlating to over 6,000 deaths per day. In addition, around 340 million occupational injuries and 160 million cases of work-related illnesses are registered yearly worldwide^8^. Beyond the devastating human impact, these incidents result in an estimated economic loss of nearly 4% of global GDP^9^. Despite their scale, these figures are widely considered to be underreported, specifically in low- and middle-income countries, where surveillance and management systems are weak and systemic data suppression or informal employment practices are dominant^5^. This underestimation is particularly crucial in SMEs^10^, which account for the majority of the global workforce^11^ but cannot often execute comprehensive safety measures^12^. In the European Union, for example, SMEs constitute 98% of all registered businesses and employ over 50% of the workforce^13^, yet they consistently register higher rates of occupational injuries and fatalities than larger firms^14^.

Notwithstanding their central role in economic productivity^15^, SMEs face disproportionate challenges in managing WHS^16^. These include financial^17^ and technical^18^ restrictions, the lack of proper WHS management systems^19^, insufficient training timetables^20^, lower levels of workforce representation^21^, and irregular regulatory inspections^22^. In regions affected by internal conflict or weak governance, such as parts of the middle east, these structural limitations are further exacerbated by political instability, fragmented institutional oversight, and a lack of accountability mechanisms^23^.

In post-conflict settings, workplace health and safety challenges extend beyond conventional resource limitations and are deeply embedded in disrupted institutional, legal, and governance structures^24^. Prolonged conflict often results in weakened labour inspection systems, fragmented regulatory authority, erosion of employer accountability, and limited enforcement of occupational standards^25^. In such contexts, SMEs frequently operate in semi-formal or informal arrangements, where compliance with WHS regulations is inconsistent or selectively applied^26^. Post-conflict economies are also characterised by workforce instability, skills displacement, reliance on short-term labour, and constrained public-sector capacity, all of which undermine systematic risk management and prevention efforts^27^. These conditions create a distinct WHS landscape in which traditional regulatory-driven safety models are often ineffective, necessitating context-sensitive, practice-oriented, and locally adaptable assessment approaches. Understanding WHS performance in post-conflict regions therefore requires empirical investigation grounded in the realities of institutional fragility, rather than extrapolation from stable or fully regulated economies.

The Kurdistan Region of Iraq (KRI) serves as an exact example of such vulnerable territories^28^, where small and medium-sized industries are widespread and subject to remarkable economic and political restrictions^29^. Despite sensible economic growth in recent years, the majority of small enterprises persist in operating without consistent governmental oversight or structured safety procedures^30^. Available field data and institutional assessments demonstrate that many workshops and manufacturers underreport their workforce size, limit access to official inspections, and lack even the most basic occupational safety infrastructure^31^. The prevalence of informal employment, avoidance of legal registration, and inadequate regulatory enforcement have contributed to increasingly unsafe working situations and raised rates of work-related incidents, especially in sectors such as construction, light manufacturing, and chemical processing^32,33^.

This study addresses a significant research gap by applying the ILO Ergonomic Checkpoints framework to evaluate the safety performance of SMEs in the Kurdistan Region of Iraq, a post-conflict setting where formal WHS systems are underdeveloped. Drawing on a unique dataset collected from 70 enterprises across five industrial sectors, the study offers one of the first field-based assessments of workplace safety in this region. In addition to identifying critical deficiencies in physical hazard control and emergency preparedness, it statistically explores how safety performance varies based on company characteristics such as workforce size, managerial experience, and operational maturity. These findings contribute both to the global WHS literature and to local policy efforts aiming to improve occupational safety in fragile and under-regulated environments.

Ultimately, this article proposes one of the few evidence-based, ground-level analyses of workplace safety in the region, presenting practical recommendations for nationwide labour authorities, WHS specialists, and global growth counterparts operating toward safer and more equitable industrial environments.

### Literature review

#### Global status of WHS in SMEs

SMEs form the economic backbone of most countries, including over 90% of registered businesses and using more than 50% of the global workforce^34^. However, they consistently demonstrate weaker performance in WHS compared to larger organizations^35,36^. This is largely due to restricted financial resources, informal management approaches, poor regulatory enforcement, and a lack of access to up-to-date safety infrastructure and training.

Globally, occupational injuries and diseases result in enormous human and economic losses, and SMEs are disproportionately affected. Barbosa et al. (2019)^37^ highlight that most SMEs do not possess sufficient performance indicators to measure safety outputs, which restricts their capability to react proactively to workplace hazards. Tremblay and Badri (2018)^38^ further claim that the lack of tailored assessment tools and compliance mechanisms for SMEs complicates their efforts to implement effective WHS practices, specifically in non-regulated or informal environments. Moreover, in both high-income and developing contexts, SMEs are often under-represented in national safety statistics and policy initiatives. This underrepresentation leads to systemic neglect of their demands in occupational safety reforms^39^.

Hence, there is growing consensus among global agencies that intensifying WHS in SMEs is crucial not only for workers’ well-being but also for sustainable economic development. This is extremely urgent in low- and middle-income countries where informal SMEs dominate the labour market and lack even basic workplace protections. Given these global challenges and limitations, it is essential to explore practical and accessible tools that can help SMEs improve WHS conditions, especially in resource-limited settings.

#### Application of ILO ergonomic checkpoints in SMEs

The ILO Ergonomic Checkpoints tool is widely recognized as a practical and cost-effective tool to evaluate and improve workplace safety, specifically in resource-limited backgrounds^40^. It includes 132 action-oriented guidance covering critical areas such as physical layout, material handling, machine safety, emergency preparedness, and worker training. Its modular structure and visual design allow for easy adoption in various industries, particularly in SMEs that often lack technological capability and standard safety procedures^41^.

Recent empirical studies underscore the significance of this mechanism in diverse backgrounds. For example, Pérez et al. (2021)^42^ applied the Ergonomic Checkpoints in a Colombian meat processing manufacturer and revealed measurable progress in workstation design, material storage, and lighting situations. Similarly, Kingwan et al. (2024)^43^ implemented the tool in an automotive component manufacturing SME, showing considerable improvements in posture management, and lowered musculoskeletal strain among workers. These findings underline the adaptability of the tool across different industries and geographical contexts. The instrument has also been utilised in agricultural industries, such as rubber plantations in east Kalimantan^44^ and among female farmers in Indonesia^45^. These studies show that even in low-technology settings, primary ergonomic interventions can remarkably decrease occupational risks and improve productivity when workers are actively involved in recognising problems and implementing solutions. In industrially developing countries, this tool has also been employed as a mechanism for sharing ergonomic knowledge in workplaces via participatory training sessions. For example, Abdollahpour and Helali (2022)^46^ revealed that using ergonomic checkpoints in a participatory ergonomics program enhanced hazard awareness, safety manners, and worker engagement in Iranian manufacturing firms.

Collectively, these studies demonstrate that the ILO Ergonomic Checkpoints framework is not only technically useful but also socially adaptable and flexible. It promotes employee participation, facilitates risk identification, and supports incremental safety modifications even in inadequately regulated conditions, a key part of its successful usage in fragile or post-conflict territories. While these case studies demonstrate the potential of ergonomic checkpoints, their implementation becomes significantly more complex in developing and post-conflict regions where systemic constraints prevail.

#### Challenges in implementing WHS in developing and post-conflict settings

Implementing practical WHS strategies in developing and post-conflict regions is fraught with structural, institutional, and cultural challenges. While SMEs are already known to encounter complications in embracing comprehensive safety frameworks, these challenges become even more acute in fragile settings characterized by inflexible governance, poor infrastructure, and informal labour markets. Studies such as those by Masi and Cagno (2015)^47^ and Tejamaya et al. (2021)^48^ underscore several persistent obstacles: lack of financial resources, weak enforcement of labour laws, lack of safety management systems (SMSs), and minimal worker participation in decision-making processes. In these contexts, many SMEs lack even the basic awareness of WHS obligations and legal responsibilities, resulting in low compliance and elevated accident rates.

Political fluctuation further complicates the institutional capability to regulate safety. For instance, research by Djip (2014)^49^ on post-war Bosnia and by Hearth et al. (2022)^50^ on Sri Lanka demonstrates that conflict-affected regions often encounter long disruptions in regulatory oversight, irregular inspections, and a reluctance among employers to formalize their operations due to perceived legal or tax burdens.

Furthermore, cultural and educational boundaries play a major role. In many developing regions, the workforce often lacks proper safety training, and employers may not perceive safety as a productive investment. Unnikrishnan et al. (2015)^51^ underscore that in the lack of rapid economic returns, many SMEs deprioritize WHS despite the long-term costs of accidents, absenteeism, and low morale. Moreover, high levels of casual employment in developing economies lead to employees being excluded from protections afforded by national legislation. This issue is well established in the Kurdistan Region of Iraq and other parts of the Middle East, where SMEs dominate the economy but evade formal registration and safety compliance mechanisms^52,53^.

Therefore, managing WHS in post-conflict and developing countries, needs more than specialised mechanisms, it demands institutional strengthening, targeted policy interventions, and culturally sensitive training programs tailored to the socio-economic facts of these areas. To overcome these obstacles, context-sensitive and participatory strategies are increasingly being recognized as effective pathways for enhancing WHS in SMEs.

#### Strategies and participatory approaches in enhancing WHS in SMEs

To address the structural and institutional limitations encountered by SMEs, particularly in developing or fragile regions, researchers and international bodies have advocated for participatory, low-cost, and context-specific interventions^54^. A notable illustration is the usage of the ILO Ergonomic Checkpoints, which highlight simple, low-investment changes that enhance safety, amenity, and productivity^40^.

Participatory ergonomics^55^, which actively implicates employees and managers in identifying risks and designing modifications, has been confirmed to be a prosperous approach in diverse SMEs. Studies such as those by Abdollahpour and Helali (2022)^46^ and Pérez et al. (2021)^42^ indicate that the ergonomic checkpoints tool can stimulate practical improvements even in resource-constrained backgrounds by translating complex ergonomic principles into easy-to-understand actions. These approaches are effective because they do not require high levels of technical expertise or costly tools. For example, Restuputri et al. (2023)^45^ revealed how simple ergonomic interventions such as improving posture or classifying tools more effectively resulted in substantial declines in discomfort and productivity losses among women farmers in Indonesia. In addition, Sohrabi (2019)^56^ used low-cost ergonomic interventions in Iranian handicraft workshops and discovered that even minor adjustments, when executed via a participatory procedure, especially decreased musculoskeletal complaints and fatigue.

These successes are not only anecdotal but reflect a broader trend in the literature, empowering workers to contribute to WHS modifications improves both safety developments and organizational buy-in. This aligns with Kogi’s (2007)^57^ findings that action-oriented training based on ergonomic checkpoints encourages a culture of prevention and joint responsibility, particularly in settings where top-down regulation is inefficient or missing. Therefore, evidence extremely supports that participatory ergonomics, especially through tools like the ILO Ergonomic Checkpoints, is one of the most practical, scalable, and flexible approaches for enhancing workplace safety in SMEs, particularly within the context of developing and post-conflict settings. Despite these encouraging efforts, significant research gaps remain, particularly in evaluating the effectiveness of such approaches in fragile and post-conflict environments.

### Research gaps

Despite the growing body of literature on WHS in SMEs, several key gaps remain, especially about fragile, post-conflict, or under-regulated regions. The majority of studies utilising tools like the ILO Ergonomic Checkpoints have been conducted in relatively sound and structured backgrounds, such as Colombia^42^, Indonesia^44,45^, or Malaysia^58^, where institutional support and essential regulatory management exist. However, very few studies have developed this framework for real-world settings influenced by political instability, informal labour markets, and weak governance, such as Iraq, Syria, or parts of Sub-Saharan Africa.

Moreover, while past studies have underscored the obstacles to executing WHS systems in SMEs, including cost limitations, lower awareness, and insufficient training^38,47^, less attention has been paid to how ergonomic-based evaluation tools can be systematically deployed in such restrained settings to create actionable safety insights. In addition, although participatory ergonomics is widely advocated, many studies assessing its influence have concentrated on personal interventions or single-case contexts, with unreasonable comparative analysis across multiple industrial sectors. There remains an absence of empirical, field-based studies that develop large, structured datasets assessing safety performance across various SME types in fragile economies.

This study seeks to address these gaps by running one of the first extensive applications of the ILO Ergonomic Checkpoints framework in the KRI, a region characterized by informal employment, limited state capability, and high exposure to occupational risks. Throughout collecting and analysing data from 70 SMEs across five industrial sectors, this study provides a novel contribution to the literature: Accordingly, this study aims to: (i) quantify WHS performance using a globally recognized framework, (ii) identify sector-specific strengths and weaknesses, (iii) examine correlations with firm-level variables such as workforce size and managerial experience, and (iv) offer practical, data-driven recommendations tailored to post-conflict environments.

As such, this study not only develops the methodological utility of ergonomic checkpoints into underexplored geographies but also fills a crucial knowledge gap in the discourse on WHS in SMEs operating under fragility.

## Methods

### Study design and setting

This research utilised a cross-sectional and observational field-based design to assess the WHS performance of SMEs in the Raparin Administration, a district within the Sulaymaniyah Governorate of the KRI. The study received ethical approval from Tabriz University of Medical Sciences and administrative consent from relevant regional bodies, which facilitated field access and coordination.

The study concentrated especially on SMEs due to their majority in the region and their known vulnerability to occupational hazards in post-conflict and under-regulated environments. This study employed the ILO Ergonomic Checkpoints framework as the main assessment instrument. Its selection was based on its practicality in resource-constrained industrial settings and its established effectiveness in evaluating safety in small-scale enterprises.

**Fig. 1:**
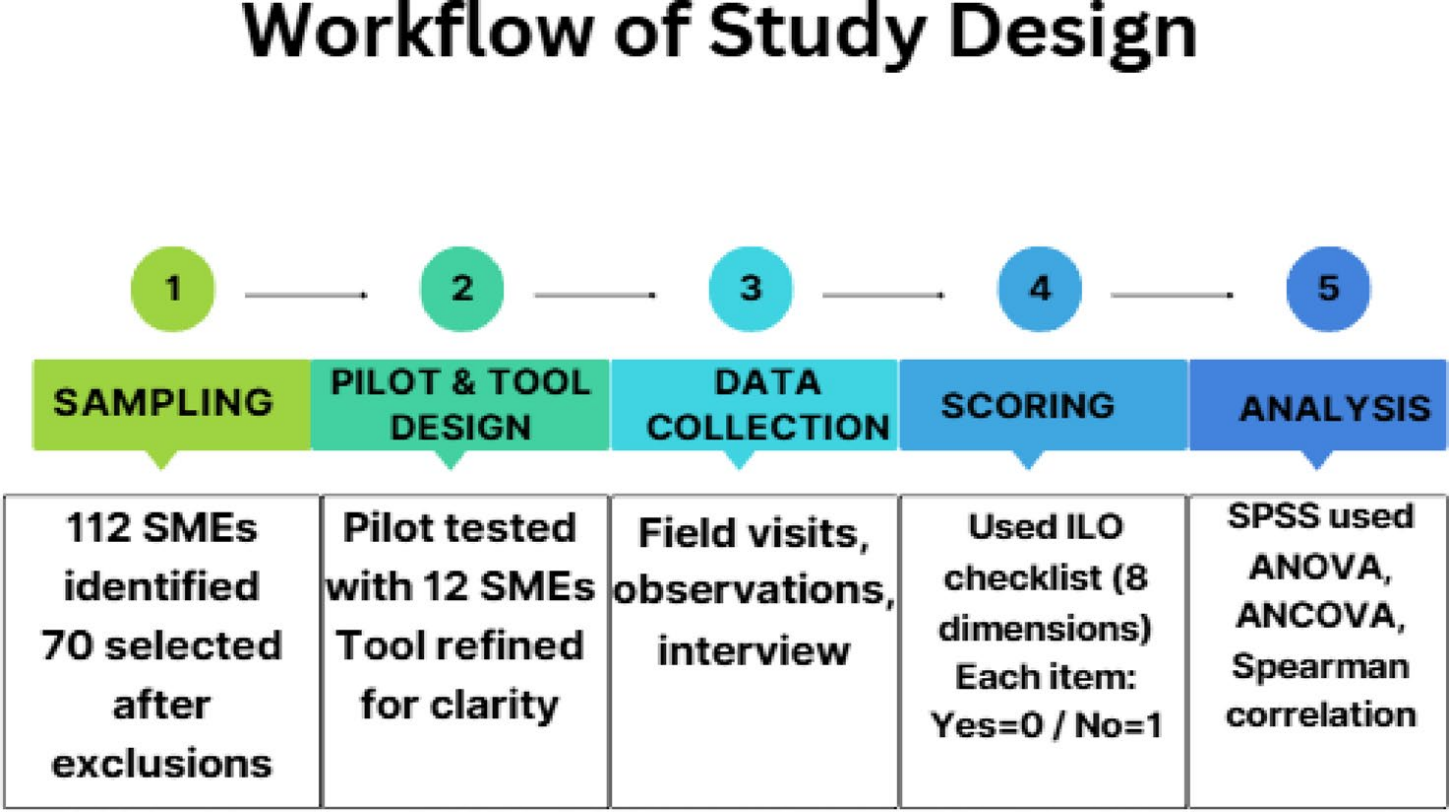
Workflow of field study on occupational health and safety in SMEs.

The Fig. 1 summarizes the five key stages of the research process: initial sampling, pilot testing and refinement of the instrument, on-site data collection, scoring using the ILO Ergonomic Checkpoints framework, and final statistical analysis using SPSS (ANOVA, ANCOVA, and Spearman’s correlation).

All ethical procedures were rigorously observed. The study protocol acquired formal approval from the Ethics Committee of Tabriz University of Medical Sciences. Further, the project obtained official administrative approval from both the University of Raparin and the Directorate of Industrial Development in Sulaymaniyah, which streamlined access to enterprise documents and field coordination. All methods were carried out in accordance with the relevant guidelines and regulations approved by the Ethics Committee of Tabriz University of Medical Sciences. All study procedures were performed according to relevant guidelines and regulations. Verbal informed consent was obtained from all participants before data collection, following ethical approval granted by the Research Ethics Committee of Tabriz University of Medical Sciences.

### Study sample and participants

This study targeted SMEs operating in the Raparin district of the Kurdistan Region of Iraq. A complete list of registered enterprises was obtained to define the sampling frame. The target sample for this study consisted of all officially registered SMEs operating within the Raparin Administration, Sulaymaniyah Governorate, in the KRI. A comprehensive sampling frame was received from the Directorate of Industrial Development, which presented a verified list of 112 registered SMEs in the study region at the time of data collection. Among the 112 registered SMEs, 42 were excluded due to inactivity or refusal to participate, resulting in a final sample of 70 eligible enterprises across five industrial sectors.

Eligible enterprises were defined as those with fewer than 100 employees, located within Raparin, and formally registered with the Kurdistan Labour Syndicate. Enterprises not meeting these criteria or those declining participation were excluded.

A pilot study was conducted prior to the main data collection phase. Its aim was to assess the clarity and relevance of the structured questionnaire, as well as to evaluate the average time required for its completion. This initial testing involved 12 SMEs, chosen from the same sampling frame, and took place under field conditions like those in the final study. The pilot demonstrated that, on average, it took about five hours per enterprise to complete all sections of the questionnaire, including workplace walkthroughs and interviews with management and workers. Based on pilot feedback, minor revisions were made to improve the clarity of a few items, especially in the ergonomics and emergency preparedness sections.

### Data collection Instruments

The identification of factors and the development of the data collection instrument followed a structured, evidence-based process informed by international guidelines and contextual considerations. Initially, core workplace health and safety domains were defined using the ILO Ergonomic Checkpoints framework, which provides a validated and action-oriented structure for assessing WHS conditions in small and medium-sized enterprises. Questionnaire items were selected and adapted based on their relevance to SME-scale operations, observability during on-site field assessments, and applicability to post-conflict and resource-constrained industrial environments. In parallel, organizational, and contextual variables were included based on their theoretical relevance and repeated use in prior WHS studies, particularly factors related to enterprise structure, managerial capacity, and workforce characteristics. This approach ensured that the instrument systematically captured both ergonomic safety conditions and underlying organizational determinants of WHS performance.

The questionnaire consists of two main sections, containing a total of 88 items, and was developed to seize both organizational context and workplace safety performance:

#### Section 1: socio-demographic and company characteristics (28 items)

This section collected detailed contextual data on each enterprise, including:

- Workforce design: total number of employees, number of male and female workers, and their age.
- Wage system: average monthly wage (in Iraqi Dinar).
- Years in operation and managerial experience of top executives.
- Existence of WHS budgets and allocation of resources for it.
- The existence or lack of documented SMSs and safety specialists.
- Documented annual workplace incident reports, fire control systems, and emergency response plans (ERPs).

These variables were employed to explore associations between organizational characteristics and safety performance outcomes across the sample.

#### Section 2: ergonomic safety assessment checklist (60 items)

This section operationalized the ILO Ergonomic Checkpoints into eight thematic dimensions suitable to WHS. The designated 60 items were adapted from the original set of 132 checkpoint recommendations and structured as follows:

1. Material handling (6 items): Evaluating lifting techniques, transportation paths, and handling equipment.
2. Hand tools (6 items): Assessing design suitability, ergonomic fit, and maintenance status of manual tools.
3. Machinery safety (20 items): Covering equipment safeguarding, maintenance, emergency stop mechanisms, and operator visibility.
4. Physical hazards (3 items): Measuring exposure to noise, temperature extremes, and poor lighting.
5. Emergency preparedness (5 items): Assessing the availability of evacuation procedures, fire alarms, and safety signage.
6. Worker participation (9 items): Gauging involvement in safety decisions, reporting systems, and feedback mechanisms.
7. Safety training (6 items): Measuring frequency, content quality, and accessibility of safety-related training.
8. Work assignment (5 items): Reviewing job design, workload balance, and rotation policies.

Each item was scored using a binary scale:

- “Yes” (compliance with the recommended checkpoint) = 0 points,
- “No” (non-compliance or absence of measures) = 1 point.

Therefore, the total safety score for each enterprise ranged from 0 (fully compliant) to 66 (maximum non-compliance), with higher scores indicating poorer safety performance.

The bilingual questionnaire (English–Kurdish) was piloted in 12 enterprises and revised for clarity and cultural adaptation. Instructions were homogenised to provide consistency in administration, and responses were verified through on-site walkthroughs, managerial interviews, and worker observations, improving the tool’s validity and reliability under field circumstances.

### Instrument reliability and internal consistency

To evaluate the internal consistency and reliability of the ergonomic safety assessment tool, Cronbach’s alpha coefficients were calculated for each of the eight dimensions of the ILO Ergonomic Checkpoints checklist. As shown in Table 1, the Cronbach’s alpha values ranged from 0.72 (Physical Hazards) to 0.88 (Machinery Safety), with an overall alpha coefficient of 0.91 for the full 60-item checklist, indicating excellent internal consistency.

Most dimensions exceeded the reliability threshold (α ≥ 0.70), as detailed in Table 1. The slightly lower alpha value for Physical Hazards (0.72) is acceptable, given the limited number of items in that category (*n* = 3).

**Table 1:** Internal Consistency and Score Range of the Ergonomic Safety Dimensions.

Construct	No. of items	Possible score range	Cronbach’s alpha	Acceptable threshold	Interpretation
Material handling	6	0–6	0.78	≥ 0.70	Acceptable
Hand tools	6	0–6	0.81	≥ 0.70	Good
Machinery safety	20	0–20	0.88	≥ 0.70	Good
Physical hazards	3	0–3	0.65	≥ 0.70	Marginal
Emergency preparedness	5	0–5	0.73	≥ 0.70	Acceptable
Worker participation	9	0–9	0.76	≥ 0.70	Acceptable
Safety training	6	0–6	0.80	≥ 0.70	Good
Work assignment	5	0–5	0.79	≥ 0.70	Acceptable
Total checklist score	60	0–60	0.91	≥ 0.70	Excellent

### Data collection procedure

Data collection was completed over one year by the researchers through direct field assessments, integrating structured walkthrough observations, semi-structured interviews, and guided checklist applications within the operating premises of each participating enterprise. The data collection procedure was carefully developed to secure consistency, contextual relevance, and reliability of results, and was conducted in alignment with ethical protocols endorsed by the Research Ethics Committee of Tabriz University of Medical Sciences.

A structured six-phase protocol guided the fieldwork, based on the implementation methodology outlined in the ILO Ergonomic Checkpoints manual. Prior validation was conducted via a pilot study to ensure methodological appropriateness.

#### Preliminary site engagement

Researchers initiated site visits with short meetings involving enterprise managers or WHS officers to identify key operational zones, gather contextual data on workforce structure and processes, and perform exploratory walkthroughs.

#### Checklist-based assessment

The 60-item ILO Ergonomic Checkpoints checklist was administered through direct observation and structured interaction with workers and supervisors. Each item was scored in real time to reduce recall bias.

#### Feedback and data documentation

Following the assessment, researchers conducted brief group feedback sessions to validate findings and suggest ergonomic improvements. Data were then cleaned, coded, and entered into SPSS for analysis, ensuring anonymity and ethical compliance.

### Data entry and scoring

All collected data were coded and entered into SPSS Version 25. To ensure accuracy and completeness, a random double-entry verification process was performed on a subset of questionnaires, minimizing transcription errors and validating data integrity. The scoring procedure for ergonomic safety performance was based on the binary coding scheme derived from the ILO Ergonomic Checkpoints methodology. Specifically:

- A response of “Yes” (meaning compliance with the recommended ergonomic or safety measure) was scored as 0, reflecting no violation.
- A response of “No” (meaning lack of compliance) was scored as 1, denoting the presence of a safety deficiency.

To enable comparative analysis across industrial sectors and organizational characteristics, sub-scores for each of the eight ergonomic dimensions were also calculated. These sub-scores allowed the researchers to identify sector-specific weaknesses, detect patterns of recurrent violations, and explore correlations with enterprise-level variables (e.g., company size, workforce age, managerial experience).

In cases where responses were missing or ambiguous, they were treated conservatively as non-compliant (score = 1) only if observational or interview-based evidence supported the absence of the required ergonomic measure. Otherwise, the item was excluded from the total score denominator for that enterprise to maintain the robustness of the analysis.

This scoring protocol ensured that the assessment was both quantitatively rigorous and contextually sensitive, capturing the degree of ergonomic compliance in a consistent and interpretable manner across a heterogeneous sample of SMEs in a post-conflict setting.

### Statistical analysis

All statistical analyses were performed using IBM SPSS Statistics Version 25. The analysis followed a structured, multi-stage process to both describe the data and examine relationships between variables of interest.

To explore group differences and predictive associations, one-way ANOVA, ANCOVA (adjusted for five covariates: managerial experience, worker age, average wage, workforce size, and company age), and Spearman’s rank-order correlation were used. Effect sizes (partial eta squared, correlation coefficients) were calculated where applicable. Model assumptions, including homogeneity of variance (Levene’s test), normality of residuals (Shapiro-Wilk and Q-Q plots), and multicollinearity (VIF), were tested and satisfied. The Shapiro–Wilk test was used to assess the normality of the total safety score distribution. The results indicated that the normality assumption was not fully satisfied, which informed the use of Spearman’s correlation for examining associations between continuous variables. Nevertheless, ANOVA and ANCOVA were retained for group comparisons due to their robustness to moderate deviations from normality and the exploratory nature of the analysis.

This approach tested whether mean safety scores significantly differed among the five groups. To control for the effect of potential confounding variables, including managerial experience, average worker wages, workforce size, mean worker age, and years since establishment, a one-way analysis of covariance (ANCOVA) was conducted. This allowed for a more accurate estimation of sector-specific effects on safety performance, accounting for organizational variability. To further analyse relationships between continuous organizational characteristics and overall safety scores, Spearman’s rank-order correlation was employed. This non-parametric method was chosen due to its robustness in handling non-normal distributions and ordinal relationships. All statistical tests were two-tailed, and results were assessed as statistically significant at a p-value less than 0.05. A 95% confidence interval (CI) was employed to analyse the accuracy of the calculations.

Where suitable, effect sizes (partial eta squared for ANCOVA, correlation coefficients for Spearman’s test) were also calculated to assess the magnitude of observed associations. Assumptions of each statistical test, including homogeneity of variances for ANOVA and linearity for correlation, were checked and reported. This comprehensive statistical approach allowed for both group comparisons and correlational inferences, enabling the identification of sector-specific safety gaps and the influence of organizational features on workplace safety compliance. The combination of ANOVA, ANCOVA, and correlation analysis was intentionally employed to address complementary analytical objectives. One-way ANOVA was used to examine whether mean workplace safety scores differed significantly across industrial sectors. ANCOVA was subsequently applied to assess sectoral differences while statistically controlling for potential confounding organizational variables, including managerial experience, workforce size, average wage, and years of establishment. Finally, Spearman’s correlation analysis was used to explore the strength and direction of associations between continuous organizational characteristics and overall safety performance. Together, these methods allowed for group-level comparison, covariate-adjusted inference, and relational analysis, providing a comprehensive understanding of both structural differences and organizational determinants of WHS outcomes.

Prior to conducting the ANCOVA, all key assumptions were examined. The normality of residuals was assessed using Shapiro-Wilk tests and Q-Q plots. Homogeneity of variances was tested via Levene’s test, and the assumption of homogeneity of regression slopes was evaluated by testing for interaction effects between the covariates and the independent variable. No violations of these assumptions were detected. For the Spearman’s rank-order correlation, since the method is non-parametric, it inherently accounts for non-normality and ordinal-scale data, but scatterplots and monotonicity were visually inspected to confirm the suitability of the test.

## Results

Before performing the one-way ANOVA and ANCOVA, essential statistical assumptions were conducted to ensure the validity of the models. The homogeneity of variances was evaluated using Levene’s test and confirmed to be non-significant across all dependent variables (*p* > 0.05). As shown in Table 2, the p-values for all dimensions were greater than the 0.05 significance level, revealing no statistically significant differences in variance across the industrial sectors. Therefore, the assumption of homogeneity of variance was satisfied for all dependent variables. This demonstrates the suitability of employing one-way ANOVA and ANCOVA in the comparative analyses performed in this study.

**Table 2:** Levene’s test for homogeneity of variances across ergonomic dimensions and total safety score.

Dimension	Levene’s F	*p*-value	Variance homogeneity
Material handling	1.12	0.35	Assumed
Hand tools	0.96	0.42	Assumed
Machinery safety	1.45	0.22	Assumed
Physical hazards	2.1	0.08	Assumed
Emergency preparedness	0.88	0.49	Assumed
Worker participation	1.06	0.38	Assumed
Worker training	1.34	0.25	Assumed
Work assignment	0.79	0.53	Assumed
Total safety score	1.58	0.19	Assumed

**Fig. 2:**
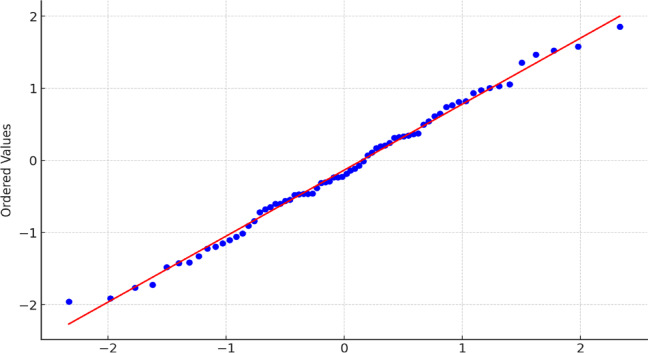
Q-Q plot of standardized residuals demonstrating approximate normality.

The quantile–quantile (Q-Q) plot presented (Fig. 2) above visually compares the distribution of standardized residuals against a theoretical normal distribution. Most data points lie closely along the 45-degree reference line (shown in red), indicating minimal deviation from normality. This visual evidence supports the assumption of normally distributed residuals, which is critical for the validity of parametric analyses such as ANOVA and ANCOVA employed in this study. Minor deviations observed at the distribution tails are within acceptable bounds and do not materially affect the robustness of the inferential tests.

**Fig. 3:**
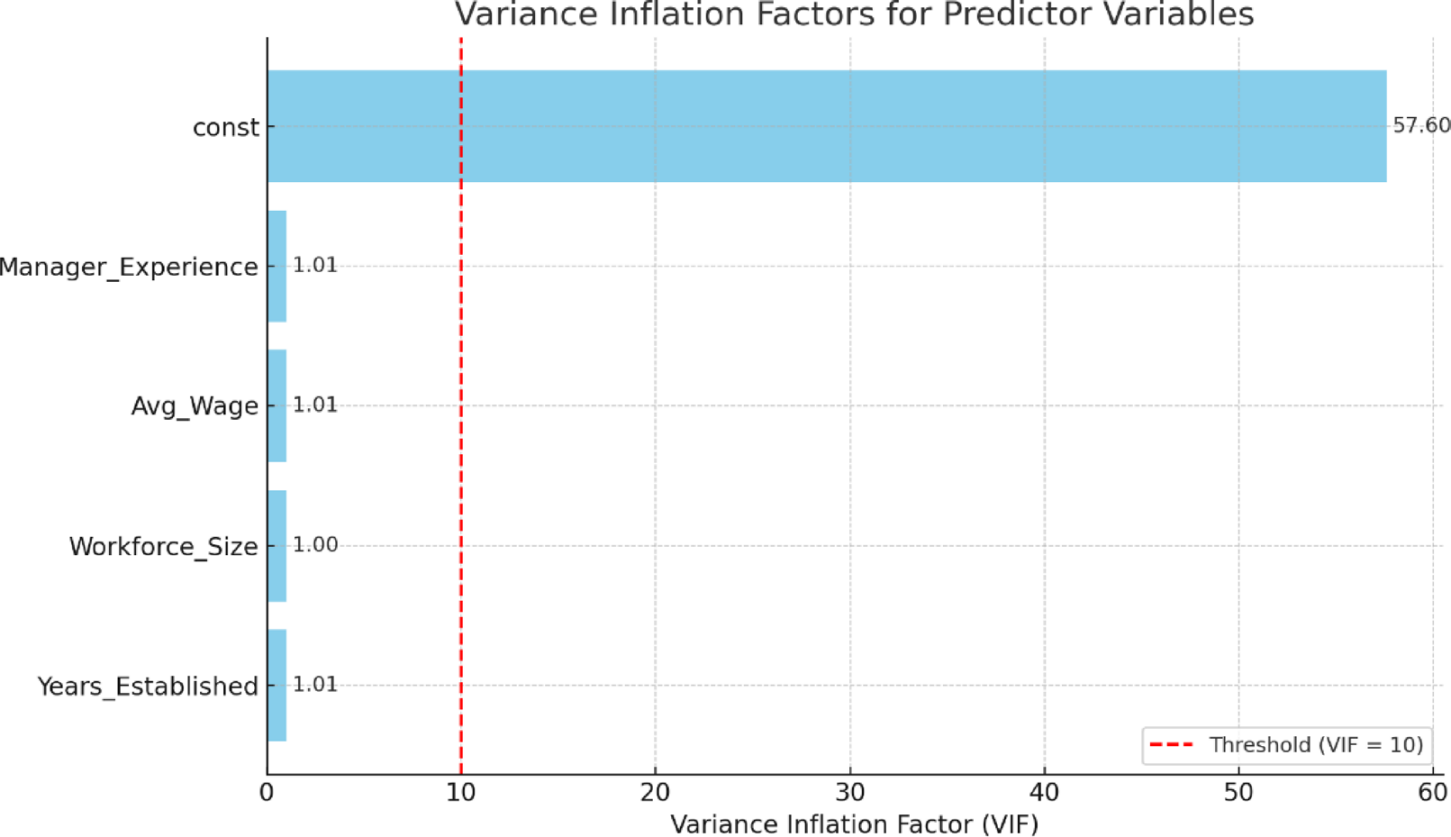
Variance inflation factors (VIF) for predictor variables in the conceptual safety model.

The VIF analysis was conducted solely to assess the feasibility and statistical stability of the proposed conceptual model for potential future multivariate analyses. As shown in Fig. 3, all predictor variables exhibit VIF values well below the conventional threshold, indicating no multicollinearity concerns and supporting the suitability of these variables for future predictive modelling, rather than representing an empirically fitted regression model in the current study.

To ensure the validity of parametric analyses such as ANOVA and ANCOVA, the normality of residuals was statistically tested using the Shapiro–Wilk test, which is particularly suitable for small to medium-sized samples. Residuals were computed based on the difference between observed and predicted total safety scores. The test yielded a W statistic of 0.989 and a corresponding p-value of 0.791, indicating no significant deviation from normality. Therefore, the assumption of normally distributed residuals was satisfied (*p* > 0.05), supporting the appropriateness of the applied inferential analyses. Visual inspection of the Q-Q plot (Fig. 1) further corroborated this finding, as data points closely followed the reference line, suggesting an approximately normal distribution of residuals.

This study evaluated the safety performance of 70 SMEs operating in five industrial sectors in the KRI using the ILO Ergonomic Checkpoints framework. The average total safety score across all enterprises was 54.71 out of 66 (SD = 2.58) (Table 3), indicating moderate adherence to WHS guidelines. However, a breakdown of individual ergonomic domains revealed a highly uneven compliance landscape.

As shown in the following table, the highest levels of compliance were observed in the work assignment and hand tools domains, with normalized means of 91.43% and 85.71%, respectively. In contrast, physical hazards received the lowest mean compliance score of 19.29%, highlighting severe deficits in the control of environmental risks.

**Table 3:** Mean scores across ergonomic domains (*N* = 70).

Dimension	Items	Min	Max	Mean	SD	Normalized mean (%)
Material handling	6	4.00	6.00	5.66	0.51	82.86
Hand tools	6	4.00	6.00	5.71	0.54	85.71
Machinery safety	20	19.00	21.00	20.07	0.49	53.57
Physical hazards	3	1.00	3.00	1.39	0.75	19.29
Emergency preparedness	5	2.00	4.00	3.20	0.81	60.00
Worker participation	9	6.00	10.00	9.20	0.97	80.00
Worker training	6	3.00	5.00	4.57	0.65	78.57
Work assignment	5	4.00	5.00	4.91	0.28	91.43
Total safety score	60	49.00	60.00	54.71	2.58	51.95

To analyse sector-specific variations in WHS performance, a one-way ANOVA was performed to compare total safety scores across five distinct industrial categories. The examination yielded a statistically significant difference in overall safety compliance among the sectors (*p* = 0.001). Among them, service enterprises exhibited the highest average safety score (57.57 ± 2.37), indicating relatively better adherence to ergonomic and safety standards, while chemical and plastic factories declared the lowest average score (54.00 ± 2.00), reflecting greater levels of non-compliance. Notably, significant differences were also observed in most of the eight ergonomic dimensions assessed. However, emergency preparedness (*p = 0.066*) and worker participation (*p = 0.104*) did not demonstrate statistically significant variation between the groups, suggesting that these aspects may be uniformly implemented or neglected across sectors regardless of industrial type. Table 4 show the safety scores by factory type.

**Table 4:** Safety scores by factory type (Mean ± SD).

Dimension	Service	Building	Food	Construction	Chemical/plastic	*p*-value
Handling	5.14 ± 0.47	5.77 ± 0.42	5.85 ± 0.37	5.80 ± 0.42	5.00 ± 0.00	0.001
Hand tools	6.00 ± 0.00	5.52 ± 0.64	5.85 ± 0.37	6.00 ± 0.00	6.00 ± 0.00	0.015
Machinery safety	20.71 ± 0.48	20.00 ± 0.00	19.14 ± 0.37	20.00 ± 0.00	21.00 ± 0.00	0.001
Physical hazards	2.71 ± 0.48	1.00 ± 0.00	2.14 ± 1.06	1.70 ± 0.82	1.00 ± 0.00	0.001
Emergency preparedness	3.71 ± 0.75	3.15 ± 0.73	3.71 ± 0.75	2.90 ± 0.89	3.00 ± 0.89	0.066
Worker participation	9.42 ± 0.53	9.00 ± 1.01	9.28 ± 1.49	9.90 ± 0.31	9.00 ± 0.63	0.104
Worker training	4.85 ± 0.37	4.40 ± 0.67	4.42 ± 0.97	5.00 ± 0.00	4.83 ± 0.40	0.039
Work assignment	5.00 ± 0.00	5.00 ± 0.00	4.85 ± 0.37	5.00 ± 0.00	4.16 ± 0.40	0.001
Total score	57.57 ± 2.37	53.85 ± 2.32	55.28 ± 2.92	56.20 ± 1.75	54.00 ± 2.00	0.001

To further examine the impact of organizational and demographic characteristics on WHS performance, an analysis of covariance (ANCOVA) was performed (Table 5). This analysis adapted for five covariates: managerial experience, average age of workers, total workforce size, average wage level, and years since company establishment. Even after controlling for these potential confounding variables, the type of enterprise remained a statistically significant determinant of overall safety performance (F (4,58) = 4.203, *p* = 0.005), indicating that differences in sectoral characteristics independently affect safety compliance levels.

Among the covariates, managerial experience emerged as a significant independent predictor of safety scores (F (1,58) = 4.818, *p* = 0.032), indicating that more experienced managers may implement or enforce ergonomic and safety practices more effectively. In contrast, other factors, including workforce size, workers’ mean age, average wage, and years of establishment, did not prove statistically significant associations with safety outcomes (all *p* > 0.05), implying a relatively weaker or indirect role in shaping safety compliance under the conditions examined.

**Table 5:** ANCOVA: total safety score controlling for demographics.

Variable	df	Mean square	F	Sig.
Factory type	4	21.452	4.203	0.005
Experience of manager	1	24.593	4.818	0.032
Workers’ wage	1	9.863	1.932	0.170
Years of establishment	1	17.328	3.394	0.071
Number of workers	1	1.946	0.381	0.539
Workers’ age	1	0.906	0.177	0.675

**Fig. 4:**
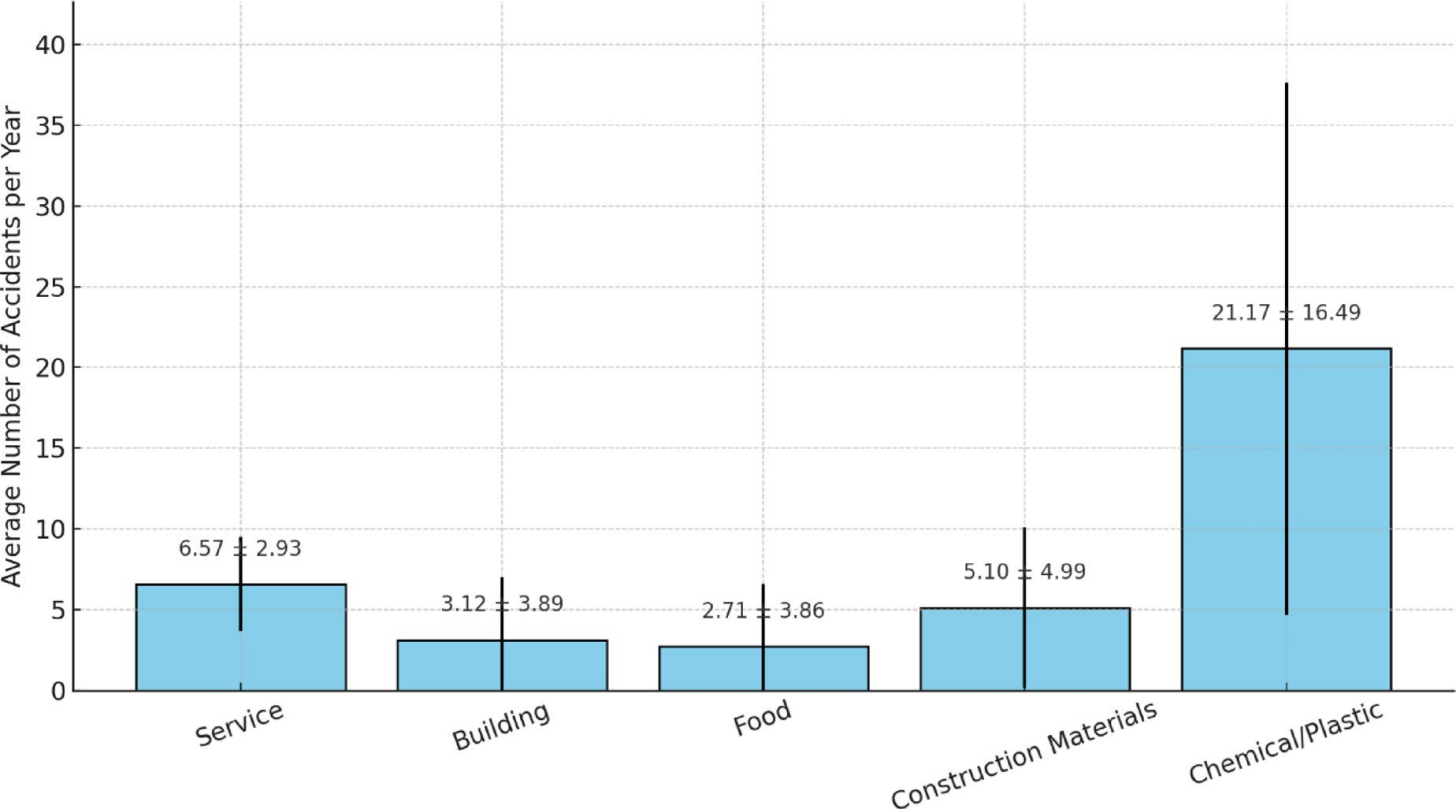
Average annual workplace accidents by industrial sector (Mean ± SD).

Significant sectoral differences were observed in the frequency of workplace accidents reported annually (Fig. 4). Notably, chemical, and plastic manufacturing enterprises exhibited a disproportionately high incidence of occupational accidents, with an average of 21.17 ± 16.49 accidents per year. This figure was substantially higher than the corresponding averages in the service sector (6.57 ± 2.93), construction (5.10 ± 4.99), building (3.12 ± 3.89), and food industries (2.71 ± 3.86). The high accident rate in chemical/plastic factories underscores the potentially hazardous nature of materials handled, suboptimal safety approaches, or deficiencies in engineering controls within this sector.

A one-way ANCOVA, adapted for relevant confounders (e.g., workforce size, manager experience, and years of establishment), demonstrated that the disparities in accident frequency across sectors were statistically significant (*p* < 0.001).

To examine the potential associations between enterprise-level organizational characteristics and overall safety compliance, a Spearman’s rank-order correlation analysis was conducted (Table 6). This non-parametric approach was chosen due to its suitability for ordinal and non-normally distributed data. The analysis investigated five continuous variables: number of workers, workers’ average age, manager’s experience, average monthly wage, and total safety score.

The results revealed no statistically significant correlations between total safety score and any of the assessed organizational variables (all p-values > 0.05). However, one noteworthy observation was a weak negative association between manager’s experience and safety score (r_s_ = − 0.198, *p* = 0.103), suggesting a possible trend where enterprises managed by less experienced supervisors may demonstrate slightly poorer safety conditions, though this relationship did not reach statistical significance.

The heatmap visually represents the Spearman correlation coefficients among workplace safety score and four key organizational factors: number of workers, average age of workers, managerial experience, and average wage. As depicted, no strong correlations were observed (|r_s_| < 0.3), but a weak negative association was found between WHS score and managerial experience (r_s_ = − 0.20). Similarly, average wage showed a slight positive correlation with safety score (r_s_ = 0.13).

**Fig. 5:**
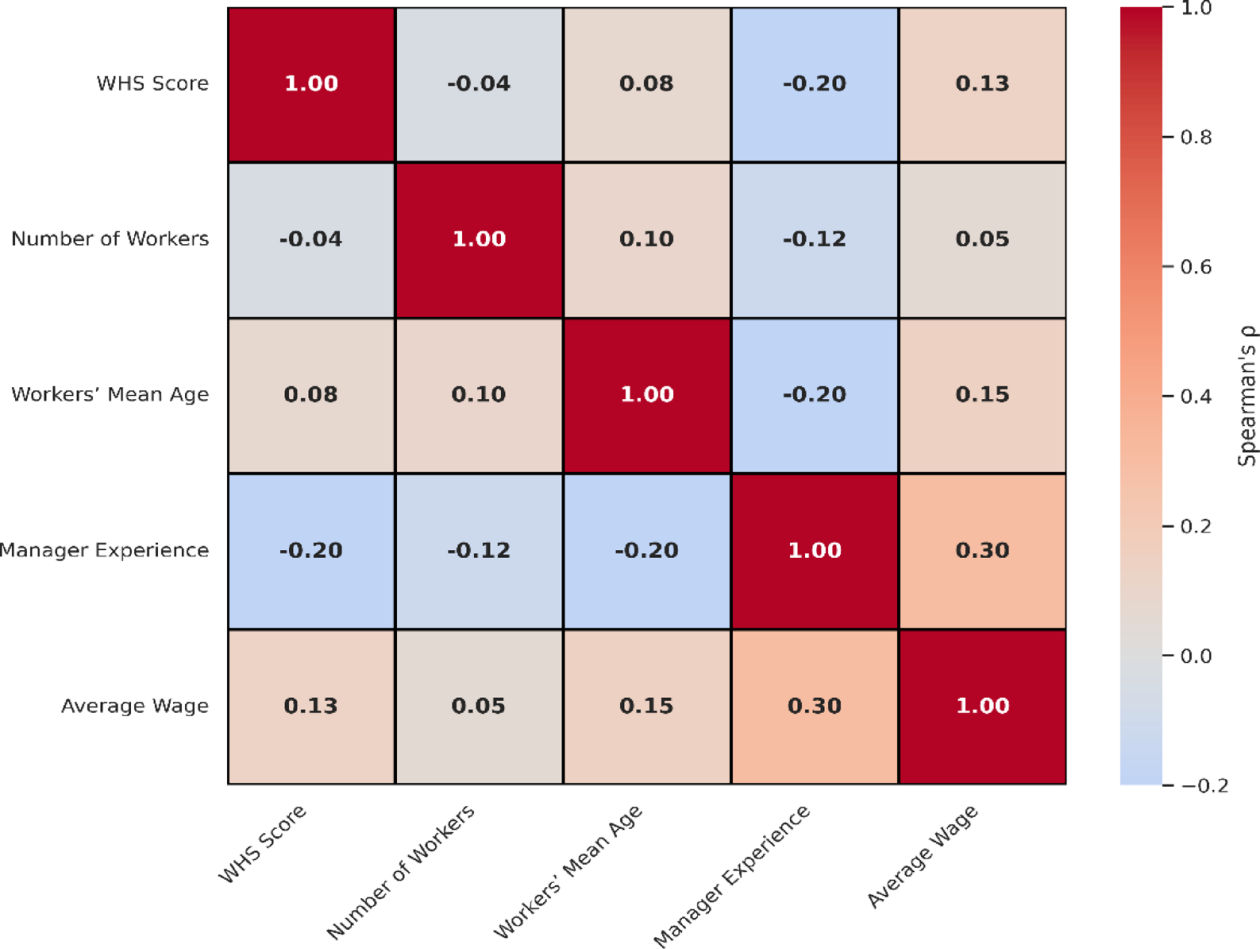
Heatmap of WHS score correlations with organizational variables.

Figure 5 lustrate heatmap diagram of WHS score correlated with organizational variables. Other variables, including number of employees (rs = − 0.039), workers’ mean age (rs = +0.080), and average monthly wage (r_s_ = +0.132), showed negligible or weak correlations with safety performance. These findings suggest that, within this sample of SMEs, safety outcomes may not be strongly influenced by basic demographic or structural factors alone.

This highlights the potential need for deeper exploration into psychosocial, managerial, and contextual determinants of workplace safety, beyond surface-level organizational metrics.

**Table 6:** Spearman’s correlation and effect size estimates between organizational variables and total safety score.

Variable	Spearman’s ρ (r_s_)	*p*-value	*R*^2^ (effect size)
Number of workers	–0.039	0.750	0.002
Workers’ mean age	+ 0.080	0.513	0.006
Manager’s experience	–0.198	0.103	0.039
Average wage (IQD)	+ 0.132	0.285	0.017

## Discussion

### Summary and interpretation of findings

This study assessed WHS performance across 70 SMEs from five industrial sectors in the KRI using the ILO Ergonomic Checkpoints framework. The average total safety score was relatively high (M = 54.71 out of 66; normalized mean = 51.95%), indicating partial compliance with WHS policies. However, substantial variation was observed across ergonomic dimensions. Domains such as work assignments and hand tools recorded elevated adherence rates (91.43% and 85.71%, respectively), demonstrating that SMEs are more capable of implementing organizational and task-based modifications demanding minimum investment. Contrarily, physical hazards and machinery safety scored the lowest (19.29% and 53.57%), suggesting systemic challenges in addressing infrastructure-heavy risks.

Statistical analysis demonstrated notable sectoral differences in total safety scores and nearly all ergonomic dimensions, with service enterprises exhibiting the highest average safety score (57.57 ± 2.37) and chemical/plastic industries the lowest (54.00 ± 2.00). These disparities were statistically significant (*p* < 0.05), validating the hypothesis that WHS adherence is extremely sector dependent. Interestingly, emergency preparedness and worker participation did not significantly differ across sectors (*p* > 0.05), which may indicate either uniformly implemented safety protocols or systemic neglect of these aspects across industries.

To address confounding variables, ANCOVA was applied using five covariates: manager experience, average wage, workforce size, workers’ age, and years established. After controlling for these factors, enterprise type remained a statistically significant predictor of safety performance (F (4,58) = 4.203, *p* = 0.005), fortifying the role of sector-specific factors in defining WHS outcomes. Among the covariates, only managerial experience showed a significant relationship with safety scores (F (1,58) = 4.818, *p* = 0.032), indicating that more experienced managers are more likely to implement effective ergonomic practices. Other variables indicated no significant effect (*p* > 0.05), suggesting that structural or demographic attributes alone may not explain safety performance variance in these settings. Moreover, Levene’s analysis for homogeneity of variances yielded non-significant findings (*p* > 0.05), confirming the hypotheses for parametric testing across all safety domains. The Shapiro–Wilk test also demonstrated the normality of residuals (W = 0.989, *p* = 0.791), which was confirmed visually through the Q-Q plot. Multicollinearity was ruled out via Variance Inflation Factor (VIF) diagnostics, with all predictor variables well below the conventional threshold (VIF < 10), thus verifying the robustness of the regression and ANCOVA models. Spearman’s correlation test further analysed the association between organizational variables and safety scores. Although none of the associations reached statistical significance (*p* > 0.05), a weak negative trend was followed between manager experience and safety score (rs = − 0.198), implying a probable inverse association under certain contextual circumstances. Overall, the lack of robust correlations supports the multifactorial nature of WHS performance, wherein cultural, behavioural, and contextual factors may outweigh purely structural ones.

Ultimately, a comparative analysis of annual workplace accidents showed alarming differences, with chemical/plastic SMEs reporting the highest frequency (21.17 ± 16.49 incidents/year), sharply contrasting with the service sector (6.57 ± 2.93). A follow-up ANCOVA verified the statistical significance of these inter-sectoral differences (*p* < 0.001), highlighting the urgency of targeted interventions in high-risk industries.

#### Conceptual implications for predictive modelling

Although the current study did not employ a standard multivariate regression analysis, it laid the groundwork for future predictive modelling by conceptualizing a theoretical framework that identifies key organizational predictors of WHS performance. Particularly, the total safety score is posited as the dependent outcome, with potential predictors including managerial experience, average worker wage, enterprise size, and years since establishment. This hypothetical relationship can be structured as:

1 $$\begin{aligned} Safety{\text{ }}Score & = \beta _{0} + \beta _{1} \left( {Managerial{\text{ }}Experience} \right) + \beta _{2} \left( {Average{\text{ }}Wage} \right) \\ & + \beta _{3} \left( {Workforce{\text{ }}Size} \right) + \beta _{4} \left( {Years{\text{ }}of{\text{ }}Establishment} \right) + \varepsilon \\ \end{aligned}$$

In Eq. (1), β₀ represents the intercept term, reflecting the baseline level of the total safety score when all predictor variables are equal to zero. The coefficients β₁ to β₄ denote the theoretical contribution of each organizational predictor to the total safety score, where β₁ corresponds to managerial experience, β₂ to average worker wage, β₃ to workforce size, and β₄ to years since enterprise establishment. The error term ε captures unobserved influences, measurement error, and contextual variability not explicitly included in the conceptual model. It represents the combined effect of omitted organizational, behavioural, and environmental factors that may influence workplace health and safety performance. This equation is presented as a conceptual framework rather than an empirically estimated regression model and is intended to guide future multivariate and predictive analyses in post-conflict SME settings. It should be noted that Eq. (1) is presented as a conceptual formulation rather than an empirically estimated regression model. Accordingly, the coefficients (β₁–β₄) are not reported in this study, as the model is intended to illustrate a theoretical structure for future predictive analyses rather than to provide parameter estimates based on the current dataset.

The presented model operates as a conceptual roadmap for subsequent empirical studies pursuing to explore the individual and combined impact of enterprise-level factors on safety outcomes. Multivariate modelling is specifically practical in such contexts because it allows the control of confounding associations and the statistical isolation of net effects within complex organizational ecosystems. For example, managerial experience may correlate with both workforce size and wage levels; a multivariate framework allows these interdependencies to be disentangled analytically.

**Fig. 6:**
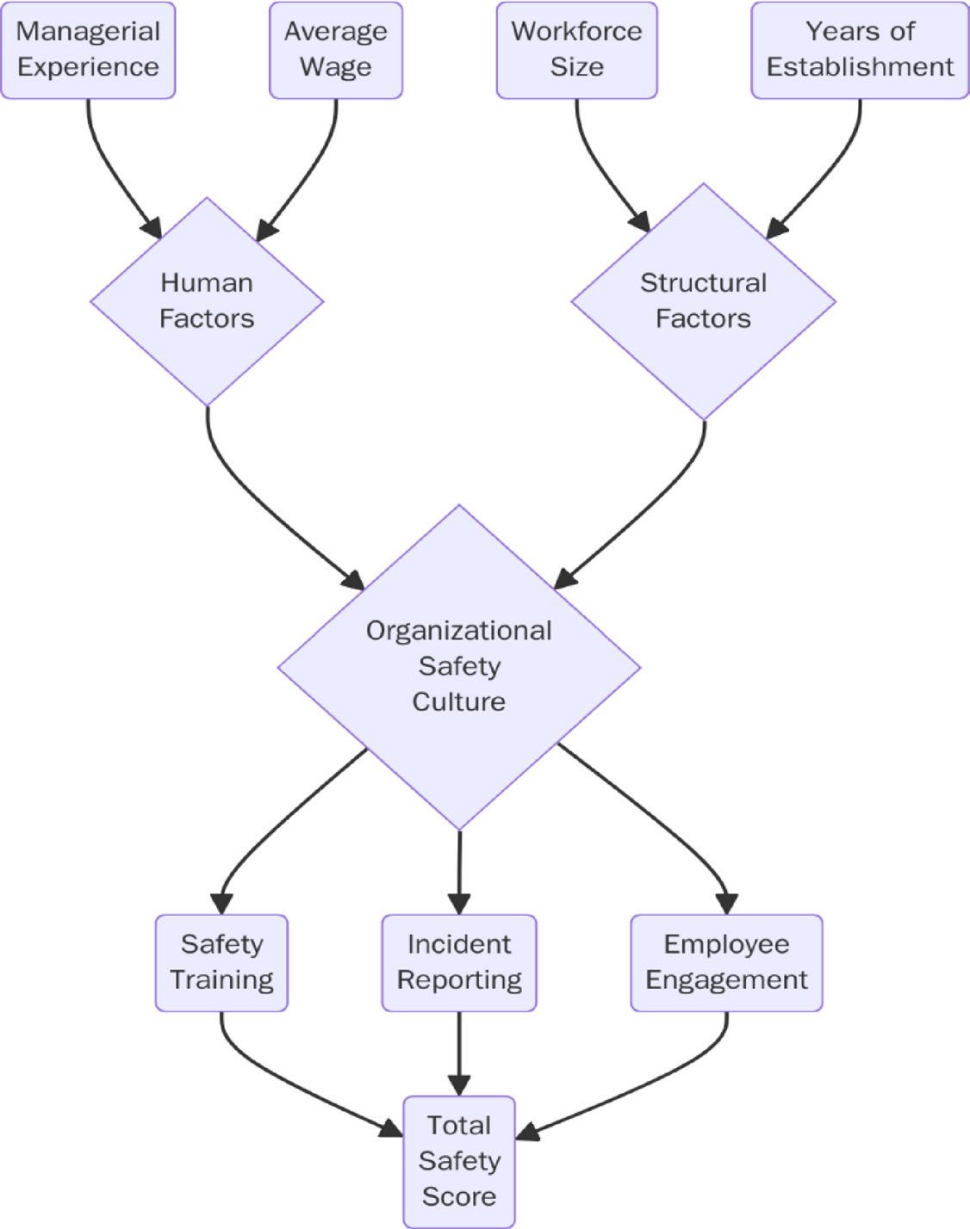
Conceptual framework linking organizational determinants to safety performance via safety culture pathways.

To enhance the interpretability and relevance of this conceptual approach, Fig. 6 shows a systems-oriented diagram that visualizes how human factors (e.g., managerial experience, average wage) and structural factors (e.g., workforce size, years of establishment) serve as upstream determinants of organizational safety culture. This cultural foundation, in turn, affects downstream mechanisms such as safety training, incident reporting, and employee engagement, each of which contributes to the overall safety performance, operationalized here as the total safety score.

This model highlights the mediating role of organizational culture in translating structural capacity and human capital into safety outcomes. This layered framework advocates methodological scalability, allowing future studies to operationalize and test the model using larger datasets, sector-specific stratifications, or longitudinal designs to better capture temporal dynamics and causal inferences.

The findings of this study provide vital insight into the state of WHS among SMEs operating in a fragile and post-conflict context and largely align with patterns documented in previous international studies. A key observation in our study was the high heterogeneity in compliance across different ergonomic domains, with physical hazards scoring the lowest (19.29%) and work assignment and hand tools scoring the highest (above 85%). This difference supports earlier findings by Abdollahpour and Helali (2022)^46^, who highlighted that while SMEs are often capable of implementing low-cost interventions related to tools and work organization, they continually lack the resources and technological capability to manage environmental and structural risks such as exposure to noise, chemicals, or poor ventilation. This is further echoed in the work of Kogi (2007)^57^ and Pérez et al. (2021)^42^, who both approve of the use of simplified and action-oriented ergonomic assessment tools as practical access points for safety modification in SMEs. Their studies indicated that small enterprises often prioritize ergonomic refinements that are visible and easy to control, while more systemic or infrastructural issues are delayed or neglected due to cost, complexity, or absence of expertise.

In terms of sectoral variation, our data demonstrated that chemical and plastic industries scored significantly lower on total safety compliance and reported the highest number of annual workplace accidents. These findings are consistent with Cagno et al. (2014)^36^ and Masi & Cagno (2015)^47^, who specified hazardous sectors such as chemicals and construction as especially vulnerable due to a mixture of higher intrinsic risk, regulatory gaps, and administrative inexperience. Their interpretive models indicate that SMEs in these sectors often operate without structured WHS management approaches, resulting in underreporting of incidents and minimal investment in prevention. Another notable result was the significant predictive role of managerial experience on safety scores, supporting statements made by Legg et al. (2015)^34^, Unnikrishnan et al. (2015)^34^, and Nowrouzi et al. (2016)^59^, who observed that supervision competence is a cornerstone of safety outcomes in SMEs. Experienced supervisors tend to implement more proactive safety practices, a pattern consistent with the observed weak negative association between managerial experience and safety scores. In contrast, workforce size, wage level, and company age showed no significant relationship with WHS performance. This aligns with the studies conducted by Tremblay & Badri (2018)^38^ and Bogna et al. (2018)^60^, who discuss that quantitative firm features are insufficient standalone indicators of WHS performance unless complemented by indicators of worker engagement, management commitment, and contextual factors.

Ultimately, this study validates the practical relevance of the ILO Ergonomic Checkpoints as a globally adaptable instrument for WHS assessment and improvement, particularly in contexts marked by informality and weak enforcement structures. Its successful application in this post-conflict setting parallels earlier interventions in agricultural, industrial, and community-based settings in Colombia, Indonesia, and Iran, as reported by Kingwan et al. (2024)^43^, Restuputri et al. (2023)^45^, and Sohrabi (2019)^56^. These outcomes underscore the tool’s versatility and reinforce the need for context-sensitive, participatory, and low-cost ergonomic interventions that empower employees and supervisors alike.

### Strengths and limitations

This study makes a novel contribution to the occupational health and safety (OHS) literature on small and medium-sized enterprises (SMEs), particularly in post-conflict and resource-limited settings. One of its main strengths lies in the use of the International Labour Organization’s (ILO) Ergonomic Checkpoints, a validated and structured tool that enables a multidimensional assessment of workplace safety, covering areas such as material handling, tool use, and worker participation. The tool was contextually adapted and combined with organizational variables to produce a localized and systems-based picture of safety conditions.

The inclusion of five distinct industrial sectors enhanced the regional generalizability of the findings. In addition, appropriate statistical methods, such as ANOVA, ANCOVA, and correlation analysis, were applied to ensure the robustness of inferences and enable a deeper exploration of the associations between organizational features and safety scores. The development of a conceptual predictive framework further positions the study as a methodological steppingstone for future research into risk forecasting and safety performance modelling.

Nevertheless, a notable limitation of this study is its cross-sectional design, which prevents any conclusions about causal relationships. While correlations between key variables were identified, causality cannot be inferred with confidence. This is a common constraint in observational research but must be acknowledged in the interpretation of results.

Despite this limitation, the combination of a theoretically grounded framework, rigorously selected tools, and robust analytical approaches lends strong methodological credibility and practical relevance to the study. The limitations stem primarily from the scope of data rather than from flaws in study design and can be addressed in future research through broader samples and longitudinal methods.

## Conclusion

This study provides a timely and evidence-based assessment of WHS states across SMEs in a post-conflict region using the ILO Ergonomic Checkpoints framework. The results underscore vital deficiencies in safety compliance, specifically within food and building sectors, and underscore the role of organizational characteristics, such as managerial experience and workforce size, in shaping safety outcomes.

By integrating ergonomic indicators with contextual enterprise data, the study presents a multidimensional view of WHS that transcends checklist reporting. Importantly, the conceptual predictive model proposed herein lays a theoretical foundation for future analytics-driven interventions aimed at improving safety intelligence in resource-limited industrial environments. Practically, the findings support targeted safety training, participatory risk assessments, and investment in human capital development as viable pathways for improving WHS outcomes. The sector-specific insights generated can inform policymakers and regulators in designing customized safety programs for vulnerable industrial domains.

While the cross-sectional nature of the data constrains causal inference, the study provides a strong methodological scaffold and an adaptable diagnostic instrument for prospective longitudinal study and predictive modelling. As the global emphasis on inclusive and sustainable work environments intensifies, context-sensitive approaches such as the one demonstrated here will be critical for enhancing safety equity and resilience in emerging economies.

## Data Availability

The datasets used and/or analysed during the current study are available from the corresponding author on reasonable request.
